# Artificial intelligence based personalized student feedback system -Sisu Athwala’ to enhance exam performance of medical undergraduates

**DOI:** 10.1371/journal.pone.0336154

**Published:** 2025-12-04

**Authors:** Thilanka Seneviratne, Supun Manathunga, Wathsala Idirisingha, Kosala Somaratne, Kosala Marambe, Udaya Dangahadeniya

**Affiliations:** 1 Department of Pharmacology, Faculty of Medicine, University of Peradeniya, Peradeniya, Sri Lanka; 2 Department of Surgery, District Base Hospital, Theldeniya, Sri Lanka; 3 Department of Medical Education, Faculty of Medicine, University of Peradeniya, Peradeniya, Sri Lanka; King George's Medical University, INDIA

## Abstract

**Background:**

In medical education, mentoring and feedback play crucial roles. Providing feedback on exam performance is a vital component as it allows students to improve. Feedback has to be tailor made and specific to the individual student. This needs lot of time and human resources, which are always not in abundance. Use of artificial intelligence (AI) is a promising proposition yet it comes with the integral problem of generating inaccurate responses by the Large language models (LLM). To alleviate and minimize this, we have developed our unique model ‘Sisu Athwala’ using retrieval augment generation (RAG) with custom LLM’s.

**Objective:**

To design and implement an AI-based tool using RAG to provide customized feedback to medical students to enhance their exam performance, minimizing the risk of generating inaccurate responses by the LLM’s. To evaluate the AI tool by expert student mentors and by the end users.

**Methods:**

The study was conducted at the Faculty of Medicine, University of Peradeniya, Sri Lanka. An AI based feedback tool was developed powered by Generative Pre-trained Transformers-4 (GPT-4) LLM using a RAG pipeline. Expert instruction sets were used to develop the data base through embedding model to minimize potential inaccuracies and biases. To generate user queries, students were provided with a self-evaluation form which was processed using Representative Vector Summarization (RVS). Hence most critical concerns of each student are distilled and captured accurately, minimizing noise or irrelevant details. The role of the AI tool was defined as a counsellor during Pre-processional alignment allowing professional manner throughout the interaction. User queries were processed using Open AI Application Programming Interface (API), utilizing GPT-4-turbo LLM. Students were invited to engage in conversations with the newly developed feedback tool. The AI tool was evaluated by the expert student mentors, as per its ability to give personalized feedback, use varied language expressions, and to introduce novel perspectives to students. End user perception on the use of AI tool was assessed using a questionnaire.

**Results:**

Post implementation end user survey of the Sisu Athwala AI tool was largely positive. 92% mentioned the advices given by the tool on stress management were helpful. 60% believed that the study techniques suggested were useful. While further 60% thought they are comfortable using the tool. 52% find the advices on exam performances were helpful. In their open comments some suggested to have the tool as a mobile APP. 15 expert student mentors took part in evaluating the tool. 100% agreed that it effectively addressed key points of student strengths and identifies areas for improvements going by the Pendleton model. 90% agreed that Sisu- Athwala gives clear actionable plans.

**Conclusion:**

Sisu Athwala AI tool provided comprehensive tailor made feedback and guidance to medical students which was well received by the end users. Expert student mentors evaluation of the material generated by the AI tool were quite positive. Though this is not a replacement for human mentors it supports mentoring to be delivered circumventing the human resource constraints.

## Introduction

The landscape of medical education is rapidly evolving, driven by advancements in technology and the increasing complexity of healthcare delivery. As medical students navigate their educational journeys, they face numerous challenges, including mastering vast amounts of information, performing well on assessments, managing stress, and developing effective study techniques [1].

Feedback serves as an essential component of education process which could minimize the gap between actual and desired outcomes [2]. The outcomes may vary by faculties, so do the feedback methods. However, certain common elements like clarity, specificity, timeliness and actionability are universal across disciplines [3].

However, implementing comprehensive feedback systems can be challenging in practical settings where large volumes of feedback must be delivered, such as in medical schools. Additionally, addressing multiple aspects of student development—including study techniques, stress management, and various social and psychological factors—within limited time constraints poses significant challenges. Traditional feedback and counseling methods often struggle to address students’ diverse needs, creating demand for innovative solutions that can provide personalized support. In this context, artificial intelligence (AI) has emerged as a promising tool to enhance educational experiences and outcomes for medical education [4,5].

The exclusive use of LLM’s such as ChatGPT in education has limitations, particularly the risk of inaccurate responses and the lack of guidance tailored to specific course content. To address these shortcomings, many recent studies have explored integrating RAG with custom LLMs.

Several developments demonstrate the application of RAG in enhancing lesson comprehension. For instance, Joseph S. Slade et al. evaluated a RAG-enhanced GPT-4 system as a tutor for introductory psychology, focusing on its effectiveness in supporting assignment writing and improving retention of course material [6]. Similarly, *OwlMentor*, an AI-powered learning environment, applied RAG to assist students in understanding scientific literature through features such as document-based chats, automatic question generation, and quiz creation. While participatory development and course integration were successful, evaluation using the Technology Acceptance Model revealed complex relationships between perceived usefulness, ease of use, and actual adoption, highlighting the need for more dynamic acceptance models [7].

Other approaches also show promise. A study in the *Eurasia Journal* reported on an LLM–RAG system designed to assess student answers for semantic correctness and deliver detailed feedback, thereby supporting comprehension of course materials [8]. Zifei et al. demonstrated how RAG-based prompting can improve the accuracy of assessing tutor competencies, enabling more effective personalized training interventions [9]. Likewise, Edison, a RAG pipeline built on GPT-4, has been developed to answer student questions with greater consistency [10]. In parallel, Seneviratne et al. introduced an AI-assisted automated short answer question (SAQ) scoring tool that uses rubrics to assess student responses, achieving performance comparable to human examiners [11].

Beyond comprehension and assessment, AI-powered chatbots are reshaping education by offering adaptive, personalized, and emotionally intelligent support. Examples include *EdgeUp*, developed by Zaryah Angels, which analyzes student performance and cognitive traits to generate structured study plans [12], and *Mindgrasp AI*, which converts academic materials into summaries, flashcards, and quizzes [13]. On the emotional support front, IBM Watson Chatbots provide conversational strategies for managing stress and anxiety [14], while *QANDA’s Cramify* creates customized study resources from student-uploaded content [15]. Other systems, such as *OpineBot* [16] and *AutoTutor* [17], demonstrate how LLMs can enhance feedback and adapt to both cognitive and emotional states during learning interactions.

Despite significant advances in AI-driven educational tools, a critical gap remains in delivering high-quality, personalized feedback that addresses both academic performance and student well-being in medical education. Current systems often focus on either content comprehension or assessment automation but fall short in providing holistic, individualized guidance.

In higher education, the provision of high-quality personalized feedback remains critical to achieving academic excellence. As highlighted by Sarabipour et al. [18]. Such feedback should extend beyond academic performance to encompass effective study strategies, stress management, coping mechanisms, exam preparation, and skills in answering both multiple choice questions (MCQ) and SAQs. The central challenge is to identify student strengths and weaknesses in order to deliver individualized, actionable guidance.

In response to this need, we developed **“Sisu Athwala”**, an AI-assisted feedback system tailored for medical undergraduates. This tool provides personalized insights into MCQ and SAQ performance, stress management, and study strategies aligned with each student’s strengths and weaknesses. By offering a scalable and cost-effective approach, *Sisu Athwala* seeks to enhance learning outcomes, promote engagement, and support student well-being—addressing critical gaps in current medical education feedback practices.

In this study, we describe the development of the Sisu Athwala feedback tool, its implementation, and the evaluation of its feedback quality as assessed by expert mentors and the students who used the system.

### Objective

To design and implement an AI-based tool using RAG to provide customized feedback to medical students to address individual student performance, stress management, and study strategies.

To evaluate whether the AI-based feedback tool effectively addresses key points of students’ strengths in study performance, clearly identify weaknesses, offer insightful and novel suggestions for improvement as judged by expert mentors and to evaluate the quality and usefulness of the feedback generated by the tool, as judged by the student users.

## Materials and methods

### Setting

The study was conducted at the Department of Pharmacology, University of Peradeniya.

### Method

#### Step one - development of the AI assisted feedback tool.

The core components of this system include a LLM, which is accessed through an API. LLMs, designed to process and generate human-like text, are foundational for interpreting user inputs and generating contextually relevant responses. To support efficient and relevant information retrieval, the system employs an embedding model [19]. The embedding model transforms text data into high-dimensional numerical representations known as vectors, capturing the semantic essence of the content. These vectorized representations are then stored in a specialized vector database, which allows for the efficient querying and retrieval of relevant information based on cosine similarity for fast and accurate responses during real-time interactions. Additionally, open-source orchestration libraries are utilized to integrate these components seamlessly.

LLMs retain the knowledge on their training data encoded as weights of the model. Output from the model can reflect factual inaccuracies and biases that was originally present in training data. Additionally, the model can hallucinate, producing incorrect, yet plausible-sounding outputs. The architecture of the LLMs makes it difficult to update/revise the encoded knowledge once it is trained. RAG attempts to mitigate this problem by attaching an external knowledgebase to the model without encoding them into model weights. The external knowledgebase is first segregated into text chunks and then turned into vector embeddings. These vector representations are then stored in a vector database. At query-time, the RAG pipeline extracts the most relevant vectors to the query and incorporates the corresponding text chunks of the external knowledgebase into the prompt, which will then be processed by the LLM.

In the design of the RAG pipeline in our study, experts in the medical education field developed a structured instruction set focusing on improving MCQ marks, SAQ marks, stress reduction methods, and optimal study techniques. These instructions were refined through a detailed system message that served multiple functions: it clarified the role of the AI counselor, outlined the expected educational outcomes, and provided three concrete examples to guide users on how to interact with the system effectively. This structured instruction set was then processed through the embedding model, converting the text into vector form to allow for its efficient storage in the vector database. During the counseling process, these embedded vectors are retrieved based on their relevance to user inputs, enabling the system to provide personalized, context-aware guidance to the students.

To generate user queries, students were provided with a self-evaluation in the form of a structured questionnaire to gather information about their concerns regarding MCQ marks, SAQ marks, stress levels, and study techniques. The responses from this questionnaire were processed using a technique known as RVS [20]. RVS is a method that condenses the most salient points of the collected responses into a smaller set of representative vectors.

It is an abstractive and extractive summarization technique specifically used to distil a long context to be able to fit into the size of the context window of an LLM. It segments the text and calculates vector embeddings of each chunk and then uses k-means of clustering to cluster the vectors around a user-specified number of centroids minimizing the within-cluster sum of Euclidian distance from the centroid. Since the vector embedding captures the semantics, the text chunks corresponding to the closest vectors to the centroids are assumed to represent the concept of each cluster. Once the representative chunks are extracted, an abstractive summarization step on them will complete the summary, effectively reducing the context length significantly.

**Fig 1:**
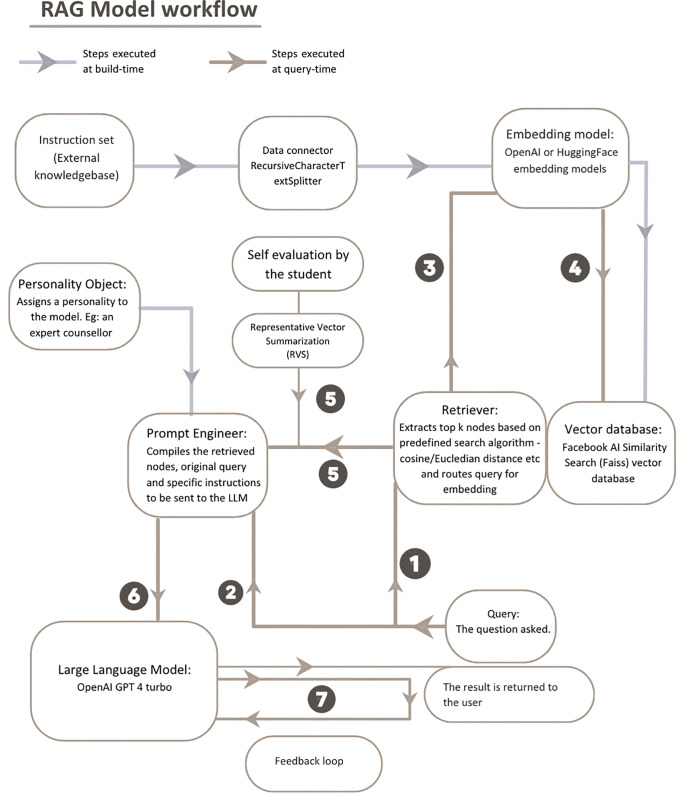
RAG model workflow.

By summarizing the key information in this way, the system ensures that the most critical concerns of each student are distilled and captured accurately, minimizing noise or irrelevant details. These summarized concerns were then mapped to pre-defined prompt templates, which are structured text frames designed to guide the formulation of the final user query, to make the input to the system clear, consistent, and tailored to elicit meaningful responses from the LLM. This user query was processed using the Open-AI API, specifically utilizing the GPT-4-turbo LLM. The AI-generated outcome underwent post-processing to structure and refine the response, ensuring clarity and relevance. (Fig 1) The system uses the docGPT retrieval workflow described in https://arxiv.org/abs/2308.00479.

Assessing the factual consistency and hallucination rate of LLMs are challenging when the training data is not publicly available. A good proxy for them would be the factual consistency and hallucination rate for summarization tasks, which allows direct comparison of model output against the source documents. This analysis is more relevant to RAG-RVS workflow in our application since in such workflows, LLMs are deployed as summarizers of the search results. Proprietary hallucination evaluation models have been used to test hallucination rates of LLMs on summarization tasks. Upon evaluating on 1000 documents from CNN and Daily Mail Corpus by the Hughes Hallucination Evaluation Model (HHEM 2.1), the OpenAI GPT 4 turbo model has shown a hallucination. Rate of 1.7% [21].

#### Step two.

Evaluation of the AI assisted feedback tool: Students were invited through student WhatsApp groups to use the AI assisted feedback tool. From 20.09 2024 to 20.10.2024.

Volunteered students were given the facility to use the feedback tool. Initially students were asked to fill a self-evaluation of their performance. This includes their study techniques, perceived stress level, coping strategies, study techniques, strengths and weaknesses. The self-evaluation of each student significantly affects the model output. Therefore, to maintain the quality and consistency, the self-evaluation sheet was designed as a structured tool with mainly close-ended questions relevant to different parts of the examination, study techniques and coping strategies.

The self -evaluation was fed into the system and then students were allowed to interact with the tool.

The output given for student queries were extracted de-identifying any personal/sensitive information. To evaluate the system’s performance this extracted output was used.

Expert human student counselors were invited to assess the quality of the feedback given by the newly developed tool.

Open invitation was sent to senior student counsellors in the faculty of Medicine via e mail. Volunteered student counsellors were provided with the extracted feedback given by the AI tool for student queries.

The AI feedback was evaluated across several key metrics and adherence to the principles of the Pendleton model of providing feedback [22]. These included the system’s ability to address the primary concerns outlined in the query, offer insightful and actionable suggestions, provide sufficient depth and detail in the feedback, and personalize the response to each student’s unique situation. Additional metrics of evaluation included the diversity of language expressions used in the AI responses and the system’s ability to introduce novel perspectives or recommendations that went beyond generic advice. The feedback from these evaluations informed iterative improvements to both the prompt design and post-processing steps.

The student counsellor’s evaluation of the extracted AI feedback was collected using a questionnaire in Likert scale as a google form.

Perceptions on the newly developed tool was collected from the students who used the tool, at the end of the conversation. The student feedback was gained using a questionnaire using a google form.

### Ethical issues

Informed written consent was obtained from the participants. Ethical clearance was obtained from the Ethical review board of the Faculty of Medicine, University of Peradeniya No. 2024/EC/26.

The information collected from this research project were kept strictly confidential. Any feedback information about students that was collected during the research were securely stored, and only the researchers had access to it. The identity of the participants were not revealed in the results. Participant anonymity and confidentiality was protected throughout. Participants had the option to delete or remove any parts of the chat history that they do not want to be exposed to others or the researchers before they access it. The feedback content given by the Sisu Athwala was extracted in the presence of the participant to ensure complete transparency. As the participation was voluntary, participants could withdraw from the study at any point. This fact was informed through the consent form.

The student perceptions and the perceptions of the expert human counsellors were collected anonymously.

## Results

### New insights gained during the development of the tool

During the design and development of the feedback system, significant new insights were gained, particularly in optimizing how the AI-generated responses could be made more personalized and relevant to each student’s concerns. One of the key learnings was the importance of refining the prompt templates and embedding techniques to capture details from student queries. The initial system iterations revealed that generic prompts often resulted in responses that lacked depth or failed to address the core concerns effectively. By implementing RVS and refining the text embedding process, the system evolved to provide more focused and meaningful answers, tailored to the individual’s specific needs. Additionally, the integration of expert feedback into the post-processing phase proved crucial in ensuring that the system output was not only clear but also actionable, helping students to better understand the strategies for improving their MCQ and SAQ performance, stress management, and study habits.

### The output of the feedback tool

The feedback process closely mirrored the steps of the Pendleton model, starting with encouraging self-assessment. For instance, when a student inquired about improving their study habits, the system initially responded with:

“You mentioned that you use spaced recall and mind maps that works well. That’s great! Can you tell me a bit more about how you use these techniques in your daily study routine?”

Evaluator responses were largely positive on this regard. All evaluators (100%) agreed that the system effectively addressed key points of students’ strengths in study performance before identifying areas for improvement. The model often followed up with positive reinforcement, like,

“Active recall can be a powerful tool in your study arsenal. Another technique you might find helpful is making short notes or summaries.”

The AI model breaks down feedback into small, manageable chunks that aligns with the Pendleton model’s structured approach of providing structured-incremental feedback. We observed that feedback to specific questions like “Can you tell me more about how you usually go about your self-study sessions?” were followed up by specific suggestions like “It seems like you might benefit from a bit more structure in your routine.” which then progress systematically, addressing different aspects of the student’s concerns step by step.

We observed an emphasis on jointly developing an action plan with the student, for example,

“You’ve mentioned challenges with managing your study schedule. Here’s a step-by-step approach that might help: Start with a weekly plan outlining your main tasks, then break these down into daily goals. Would you like to try setting up a basic schedule together?”

When appropriate, the feedback model also provided concrete actionable strategies, for example,

“Try using the Pomodoro Technique: Set a timer for 25-30 minutes, focus on your task, and take a 5-minute break after each session.” as a suggestion for a problem related to studying till exhaustion,

“Try using flashcards or mind maps to reinforce your understanding and memory of the material, especially for subjects like pharmacology.” as a suggestion for a problem related to difficulty in memorizing drug names,

“Consider creating a more structured study schedule, perhaps using planners or digital tools to organize your tasks” as a suggestion for a problem related to not maintaining a regular study routine.

The AI model also clarified certain aspects of the problems by asking cross questions in order to provide with tailored suggestions for the context, for example,

AI model: “Can you tell me a bit more about the challenges you face in keeping a consistent schedule?”

Student: “I am very tired after attending lectures and clinical”

“It’s important to find a balance that allows you to rest and recharge while still keeping up with your studies. One approach you might consider is breaking your study sessions into shorter, more manageable chunks. For example, studying for 30 minutes to an hour with short breaks in between can help maintain your focus and reduce fatigue. Have you tried any techniques like this, or do you think it might be something that could work for you?”

### Student counsellor’s assessment on the feedback given by the AI tool

15 student counsellors of various disciplines participated in the assessment.

**Fig 2:**
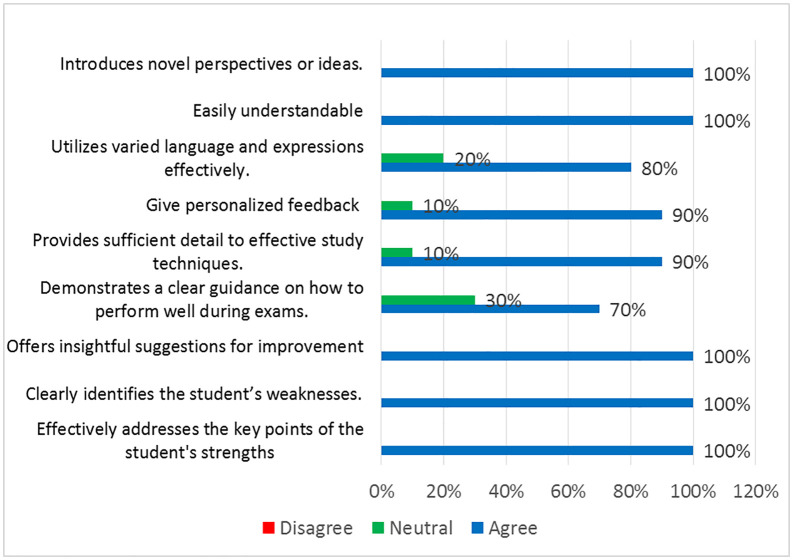
Feedback given by the student counsellors.

The majority of Student counsellors (70–90%) agreed that the system provided clear actionable plans and guidance on exam preparation, offered sufficient detail to guide effective study techniques, and personalized feedback to address individual needs and clearly identified weaknesses, and offered insightful and novel suggestions for improvement. 80% agreed that the system utilized varied language and expressions effectively for this task (Fig 2).

#### Perception of the students on the feedback received from Sisu- Athwala.

**Fig 3:**
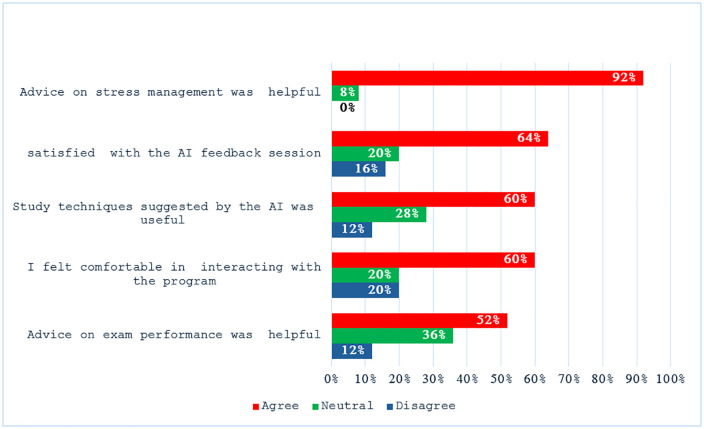
Student perception on the feedback received from the Sisu Athwala.

25 students involved in giving their perceptions on the feedback received from Sisu- Athwala**.** The AI feedback was well received by the students. Most notably 92% of students found that the advices on stress management quite helpful. 60% found that the study techniques suggested by ‘Sisu Athwala’ were useful to them. 52% found that advices given on exam performance were useful. Refer- Fig 3.

#### Open comments of the students.

Students positively appreciated the tool. And acknowledge the usefulness.

*‘it was very helpful to me’*

*‘it is really new and useful’*

For suggestions for improvements, students mentioned that feedback based on their previous exam paper performance.

*‘it is better if we could get a feedback from the papers we wrote in the last semester* ‘

The students do indicate their need to get a specific feedback on their previous exam answer sheet. This still can be accommodated in this AI model. As a development we can include the previous exam answer sheet also as one of the inputs to this model. In this being a flexible model there is a potential for further improvement.

Also student mentioned of having this feedback tool as a mobile APP.

*‘better to have a mobile app’*

It was noted that students still like human interaction and lecturer feedback. Two students mentioned that

*‘it’s better to give chance to talk with our lecturers as well. Because they absolutely know our weakness during exam and what are the methods for improving.’*

## Discussion

Several studies have explored the use of AI in educational settings. For instance, AI-driven tutoring systems have been developed to assist students in various subjects, including mathematics and language learning [23,24]. These systems typically employ rule-based algorithms or machine learning models to provide personalized instruction and feedback. Despite these advancements, existing AI systems in medical education often face limitations. Many rely on pre-defined rules and lack the flexibility to adapt to individual student needs [25]. Additionally, the feedback provided is sometimes generic and not sufficiently tailored to address specific areas of improvement. Furthermore, these systems may not adequately address the psychological aspects of learning, such as stress management and study techniques, which are crucial for medical students [26].

Traditional AI tutoring systems often provide one-size-fits-all feedback, failing to account for the unique learning needs and preferences of individual students. Many existing systems focus primarily on cognitive skills, neglecting the affective and metacognitive aspects of learning, such as stress management and effective study strategies. Rule-based systems can be rigid, offering static feedback that may not evolve with the student’s progress or changing needs. The feedback provided by traditional methods can be delayed, reducing its effectiveness in facilitating immediate learning and improvement.

### Strengths of AI-based personalized feedback system

The novel AI-based personalized feedback system- Sisu Athwala’ demonstrated promising results in providing comprehensive, tailored guidance to medical students. It was successful in providing clear actionable plans and guidance on exam preparation, offered sufficient detail to guide effective study techniques, clearly identified weaknesses and personalized feedback (Fig 2).

One of the primary limitations of AI-based chatbots is the reliability and accuracy of the information they provide. A systematic review by Mallik Salam on the utility of ChatGPT in healthcare education, research, and practice highlighted concerns such as a lack of originality, inaccurate content with the risk of hallucinations, limited knowledge, and incorrect citations, all of which were identified in 96.7% of the records [27]. This issue is especially significant when chatbots are used for providing feedback or enhancing the exam performance of medical undergraduates. Sisu-Athwala addresses this challenge by creating a database using a structured instruction set designed by experts in the field of medical education. This approach focuses on improving performance within an embedding model, ensuring high accuracy.

Despite impressive natural language capabilities of LLMs, aligning them for highly specialized tasks remains challenging. In our feedback model, we have utilized several strategies to maximize the probability of factually correct, unbiased and safe responses.

Use of verifiable external knowledge source: RAG allows us to use a curated instruction set to be used for responses, minimizing potential inaccuracies and biases of the LLM’s training dataset.

Contextualization Using Self-Evaluation Data: the model uses the student’s self-evaluation data before engaging in conversation to provide relevant feedback.

Pre-processing alignment via Personal object: The docGPT workflow includes a definition of the role to be assumed by the AI model. The workflow begins with a structured definition of the AI’s role, tone, and boundaries through a configurable “Personal” object. This instructs the model to engage in a supportive, constructive, and professional manner throughout the interaction.

Post-Processing and Feedback Loop: The final output is evaluated via a feedback loop to flag potentially harmful or inappropriate content.

Another key issue with feedback is the role of the counsellor, which goes beyond the capabilities of a chatbot. The personal object attached to the RAG pipeline describes the role the AI model should assume while carrying out the conversation. It contains instructions to clarify ambiguous inputs by asking follow-up questions. By integrating the role of an AI counsellor in the design phase, Sisu-Athwala successfully overcame this limitation, delivering clear and actionable plans.

### Ethical use of AI in academic counselling

The integration of AI into academic counselling offers opportunities for improved accessibility and support but raises significant ethical challenges. Studies indicate that students expect collaboratively developed ethical guidelines, with strong emphasis on **data privacy, explicit consent, and robust protection of sensitive disclosures** [28]. Key risks include data misuse, misinformation, over-reliance on AI, and potential undermining of deep learning and personal growth.

AI lacks empathy, emotional nuance, and moral agency, making **human oversight essential** [29]. While AI can provide cost-effective, non-judgmental interactions, especially valued by adolescents, its limitations in relational depth and ethical reflection prevent it from serving as a substitute for professional judgement. Ethical concerns also extend to **algorithmic bias, commercialization**, and inadequate professional codes that do not yet address AI-assisted care [30].

To ensure responsible use, institutions must establish **clear protocols**: safeguarding confidentiality, obtaining informed consent, transparently communicating AI’s limitations, and applying AI feedback as a supportive tool rather than a replacement for counsellors.

### AI or Human mentorship?

Concerns such as the lack of empathy, emotional nuance, absence of face-to-face interactions, are often raised when comparing AI-based feedback with human feedback. Therefore, it is important to be aware of these limitations and recognize that AI tools should be seen as complementary to, rather than a replacement for, human counsellors [31]. Studies done by Henderson et al, Zhang et al and Nazaretsky et al emphasize the importance of AI- human co- produced feedback over AI feedback alone [32–34].

This was appreciated by our student perception survey as well, as they requested the teacher feedback also to be continued hand in hand. It was interesting to see even with the huge engulf of AI in medical education, students still believe in their teachers and appreciate the AI generated tools as auxiliary to human mentorship.

### Limitations

Language barriers pose a challenge in the use of AI. For students whose first language is not English, there may be a sense of distance or discomfort when using the tool. To address this, it is crucial to explore and incorporate language translation options, enabling feedback in students’ native languages. This would enhance the tool’s effectiveness and make it more accessible for successful implementation in higher education institutions worldwide.

Despite the multiple strategies we employ to safeguard and align LLM-based systems, it is important to acknowledge a fundamental limitation: due to their design and inherently probabilistic nature, LLMs cannot guarantee complete factual accuracy or complete alignment with intended values or goals. These models generate responses based on learned patterns in vast training datasets, and even with retrieval augmentation, personal alignment, and post-processing filters, there remains a residual risk of incorrect or misleading outputs. Therefore, the model should not be viewed as replacements for human expertise or judgment, but rather as complementary tools that can enhance feedback and learning when used appropriately. Their outputs still require ongoing monitoring, validation, and auditing to ensure quality, safety, and relevance over time.

## Conclusion

Students’ feedback has to be unique specific and tailor made for the individual needs of the students. For this to happen a mentor has to spend time for the student to identify the specific needs of the student. And that is exactly where the limitations come because in any institution human resources are not unlimited.

Sisu Athwala, the personalised AI feedback tool based on RAG has answered this problem in spending time to identify the specific and unique needs of the student and giving a tailor made feedback. Which was well received by the end users. And the fact that majority of students indicating their exam related stress relaxation is addressed quite well in this model supports that conclusion.

Expert student mentors evaluation of the material generated by the AI tool were quite positive. Though this tool is not a replacement for human mentors it supports mentoring to be delivered circumventing the human resource constraints.

## Supporting information

S1 Fig Ethical clearance letter. (TIF)
