# Supplementary figures and images for: Artificial intelligence based personalized student feedback system -Sisu Athwala’ to enhance exam performance of medical undergraduates
Source: PLoS One. 2025 Dec 4;20(12):e0336154. doi: 10.1371/journal.pone.0336154 (PMC12677440; doi:10.1371/journal.pone.0336154)

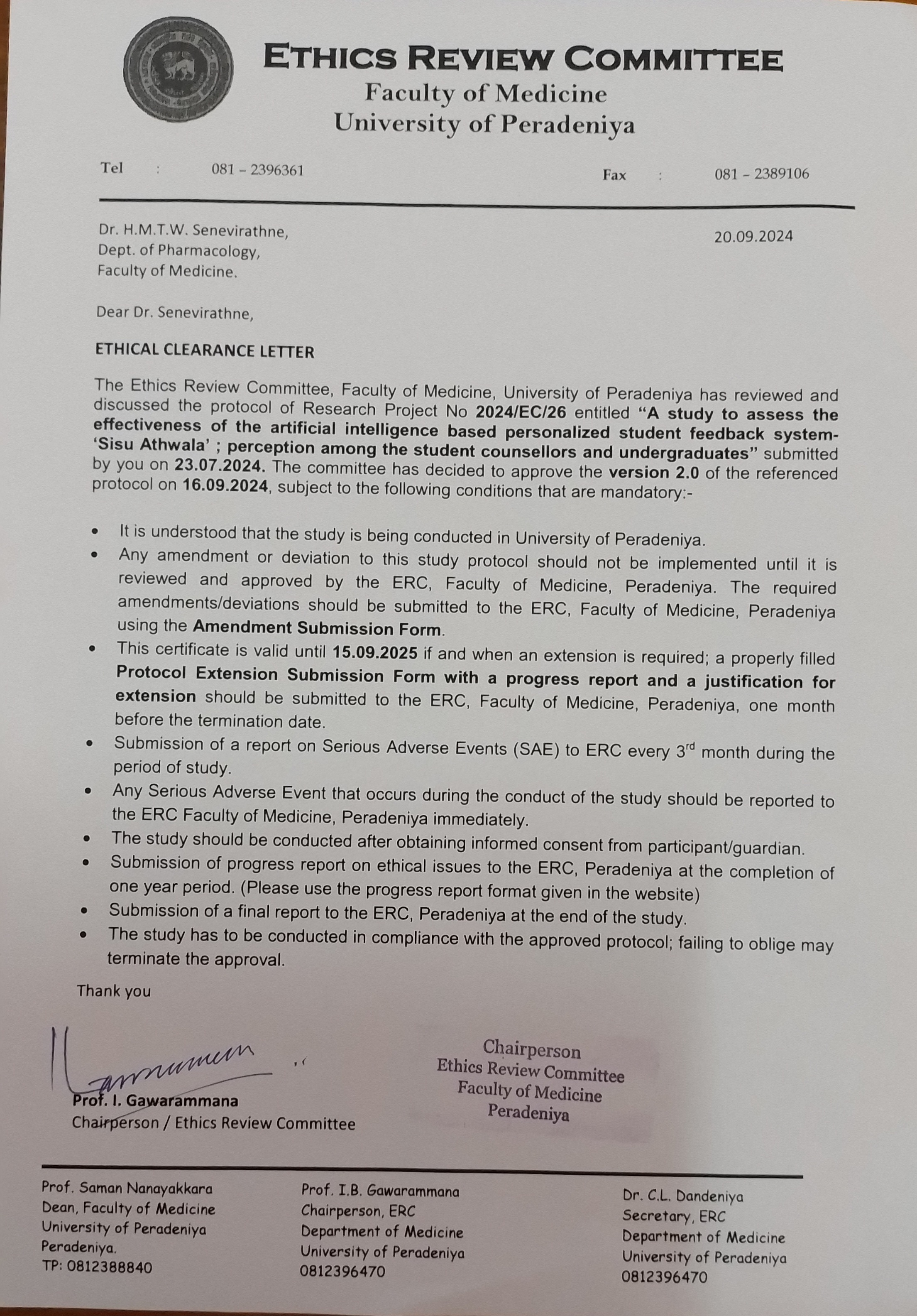

Supplement: S1 Fig — (TIF) [file pone.0336154.s001.tif]
